# Molecular analysis reveals a high diversity of *Anopheles* species in Karama, West Sulawesi, Indonesia

**DOI:** 10.1186/s13071-020-04252-6

**Published:** 2020-07-29

**Authors:** Jenna R. Davidson, Isra Wahid, Rusdiyah Sudirman, Scott T. Small, Allison L. Hendershot, Robert N. Baskin, Timothy A. Burton, Victoria Makuru, Honglin Xiao, Xiaoyu Yu, Emma V. Troth, Daniel Olivieri, Stephanny Lizarraga, Hajar Hasan, Andi Arfah, Muhammad Yusuf, Nirwana Nur, Din Syafruddin, Puji Asih, Neil F. Lobo

**Affiliations:** 1 Eck Institute for Global Health, University of Notre Dame, Notre Dame, IN 46556 USA; 2 Department of Parasitology, Faculty of Medicine, Hasanuddin University, Makassar, 90245 Indonesia; 3 Eijkman Institute for Molecular Biology, Jakarta, Indonesia

**Keywords:** *Anopheles*, Molecular identification, Sulawesi, Malaria vectors, Indonesia

## Abstract

**Background:**

Understanding local *Anopheles* species compositions and bionomic traits are vital for an effective malaria vector intervention strategy. Though eight malaria vectors, including species complexes, have been documented across the island of Sulawesi, Indonesia, a comprehensive survey linking morphological and molecular species identification has not been conducted in this global hotspot of biodiversity.

**Results:**

Eighteen distinct species of *Anopheles* were molecularly identified in a 1 km^2^ area in Karama village, West Mamuju Province, Sulawesi. Known species included *An. aconitus*, *An. karwari*, *An. peditaeniatus*, *An. vagus*, *An. barbirostris*, *An. tessellatus*, *An. nigerrimus*, *An. crawfordi*, *An. maculatus, An. flavirostris* and *An. kochi*. Of the 18 distinct sequence groups identified through both ribosomal DNA internal transcribed spacer region 2, and mitochondrial DNA cytochrome *c* oxidase subunit 1 loci, 8 could not be identified to species through comparison to published sequences. The comparison of morphological and molecular identities determined that interpretations of local species compositions for primary and expected species in Karama (*An. barbirostris* and *An. vagus*) had the highest rate of accuracy (92.1% and 87.6%, respectively) when compared to molecular analysis. However, the remaining distinct sequences molecularly identified to species were identified correctly by morphological methods less frequently, from 0 to 83%.

**Conclusions:**

Karama, Indonesia has a high diversity of *Anopheles* spp. The unexpected high number of *Anopheles* species in a small area points to possible complex transmission dynamics and limitations with vector control based on possible varying behaviors and interactions with both humans and interventions. Morphological identification of *Anopheles* spp. in this study was more accurate for primary and expected species than secondary or unexpected species. Finally, the inability to identify seven sequence groups to species with consensus sequences implies that future studies employing sequencing are required to clarify species compositions in the Nigerrimus Subgroup, among others, as well as their distribution and vector status. Use of molecular methods in conjunction with morphological investigations for analysis of species composition, population dynamics and bionomic characteristics is directly implicated in understanding drivers of malaria transmission, intervention effectiveness, and the pursuit of malaria elimination. 
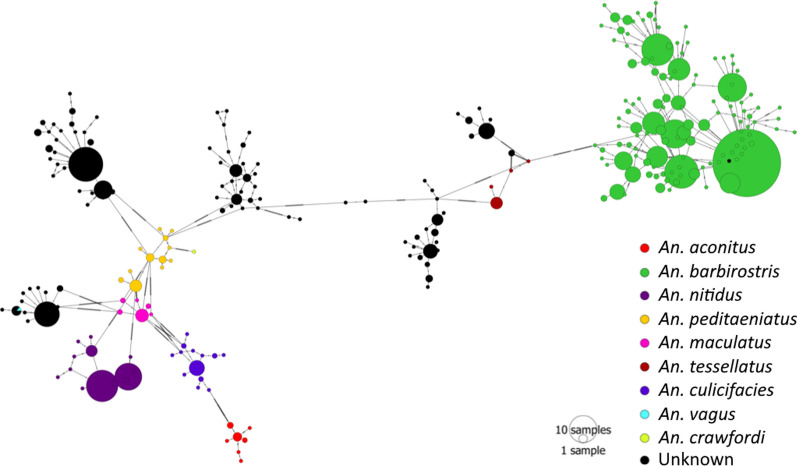

## Background

The World Health Organization (WHO) has set the ambitious goals of reducing malaria incidence and mortality by 90% by 2030 [1]. Although vector control, primarily through the use of long-lasting insecticide treated nets (LLINs) and indoor residual spraying (IRS), has led to a reduction of malaria cases worldwide [1], WHO reported a stall in progress in 2017 [2]. Despite global elimination efforts, almost half of the Republic of Indonesia’s population lives in malaria-endemic areas [3]. The highest rates of malaria cases are found in the eastern provinces of Papua, West Papua, East Nusa Tenggara, Maluku, North Maluku and Bengkulu [4, 5]. West Sulawesi has low to moderate endemicity (annual parasite index (API) of 0.99 per 1000 cases) and falls under the intensification and elimination phases of malaria control [4, 5].

Interventions target mosquito behaviors that overlap with how the intervention functions in both time and space. For example, LLINs target indoor and anthropophagic behaviors, while IRS targets indoor resting mosquitoes [6–8]. Regional or local vector populations usually consist of a variety of primary and secondary vectors, each with differing bionomic traits and seasonal population shifts, affecting the temporal and spatial protective efficacy of interventions accordingly. Data on local vector temporal composition and their behavior are crucial to the comprehension of transmission dynamics, the designing of intervention strategies, interpreting intervention efficacy, determining residual transmission, and understanding current gaps in protection from infectious bites.

Malaria is transmitted by mosquitoes of the genus *Anopheles*, which includes 475 recognized species and more un-named members of species complexes [9, 10]. Approximately 70 of these 475 species are competent as vectors of malaria parasites, while 41 species are considered dominant vectors [11]. The Indonesian archipelago has high *Anopheles* diversity, with 21 species being confirmed as vectors of malaria [5, 10–13]. Indonesia is a geographically diverse country, with differing island ecosystems, Sulawesi being a biodiversity hotspot [14]. This island, close to the Wallace line, has both Asian and Australian documented vector species and species complexes including *An. barbirostris*, *An. barbumbrosus*, *An. flavirostris*, *An. kochi*, *An. nigerrimus*, *An. parangensis*, *An. sinensis* and *An. subpictus* (*s.l*.) [12, 15–17]. There is documentation for multiple species in some complexes, such as the Barbirostris Complex [16, 18–21]. Additionally, there is molecular evidence for four cytological forms of *An. nigerrimus* in Southeast Asia [22, 23].

Published literature records indicate that multiple vectors and varied transmission dynamics are present across Indonesia [24–26] pointing to the need for a current, basic, and better understanding of species distributions, bionomic traits, and vector status, particularly relevant when considering that Indonesia aims to achieve malaria elimination by 2030 [3, 5, 10–12, 24–26].

Correct mosquito species identification is essential to understanding local mosquito species composition and associated bionomic traits that impact transmission. Identification of field-collected specimens is primarily based on morphological characteristics of adult males and females, as well as immatures [27, 28]. Although presently the most available and usually effective tool, morphological identification may be complicated by outdated, contradictory, and difficult to interpret keys [24, 26], particularly in non-African areas. Damage to crucial identifying characteristics such as the loss of scales, can occur to field-caught specimens, resulting in misidentifications. Additional issues with morphological identification include human error, presence of new or cryptic species, species with overlapping or non-documented characteristics, and intraspecific morphological variation [26]. Furthermore, accurate morphological identification requires comprehensive and rigorous training. Molecular identification allows for greater supporting granularity and may be more precise in regions of high diversity such as Southeast Asia, with an abundance of vectors, and novel, cryptic and sibling species [24–26].

To the best of our knowledge, this is the first comprehensive survey towards understanding *Anopheles* species diversity and species compositions, in and around human habitation, with molecular identification, in any area in West Sulawesi.

## Methods

### Site description

**Fig. 1:**
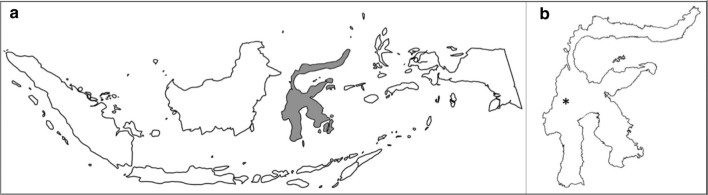
Map of Indonesia field collection sites. **a** Map of Indonesia outlining the island of Sulawesi. **b** Location of Karama village, West Sulawesi. The map was created using Google: imagery 2019 DigitalGlobe, Map data 2019

Karama, Indonesia is a single km^2^ village in the northwestern regency of Mamuju, West Sulawesi (Fig. 1). This isolated village bordered by the Sungai Karana River, is located on its flood-plain, and reaches into the foothills. The main economic activity in the area is agriculture, with the primary crop being rice. Other activities include fishing and hunting in the surrounding forest. Houses in this area are made of wood or concrete with thatched roofs. Low-lying houses are elevated on stilts due to flooding episodes. The open construction of these primarily wood houses allow for mosquito entry from all directions. This remote area has stable, year-round, malaria transmission with high incidences during the rainy season (November-March) [5].

### Mosquito collections and trap description

Mosquitoes were intermittently collected in Karama during 2013 to 2015 (Fig. 1, Table 1). Multiple sampling methods were utilized to enable the comprehensive sampling of anophelines over several years. Sampling methods included human landing catches, barrier screens, barrier screens with eaves, and kelambu traps (IW, unpublished data) [29, 30].

**Table 1:** Overview of *Anopheles* mosquito collections. Four collection methods were utilized in Karama, Indonesia during 2013–2015

Trapping method	2013			2014	2015		Total
	April–May	September	December	May	January	March	
Barrier screens	1074	566	1523	680	–	1062	4905
Barrier screens with eaves	710	–	–	–	–	1283	1993
Indoor human landing catches	1435	985	1094	1087	153	762	5516
Outdoor human landing catches	1712	1000	995	322	190	902	5121
Kelambu trap	1851	853	1486	789	126	1869	6974
Totals	6782	3404	5098	2878	469	5878	24,509

The barrier screens with eaves were not performed in the 2014 (indicated by –)

### Human landing catches (HLCS)

HLCs [29] were performed between 18:00 h and 06:00 h. Collections were performed both inside and outside houses. Collections were done in 2-h shifts, with a single collector indoors and a single collector outdoors in each sampled house (*n* = 8). After each 2-h period, the two collectors swapped positions to reduce collector bias. Location and time of collection were recorded for all mosquitoes.

### Barrier screens

Barrier screens were constructed with 2 m high, untreated bednet material secured to wooden poles at 2 m intervals for a length of 10 m. Barrier screens were set up and located as described [30]. Barrier screens were examined for mosquitoes hourly between 18:00 h and 06:00 h. Two collectors walked along each side of the trap for 15–20 min every hour, using a flashlight to spot, and mouth aspirator to collect, resting intercepted mosquitoes. Location, time of collection, and flight direction (determined by the side of the barrier screen) were recorded for all mosquitoes.

### Barrier screens with eaves

Barrier screens with eaves were constructed in the same manner as barrier screens, but with untreated bed net material eaves at the top to prevent mosquitoes from escaping over the vertical netting (NL, unpublished data). Mosquitoes were collected off barrier screens with eaves and labeled in the same manner as barrier screens.

### Kelambu traps

Kelambu traps (IW, unpublished data) are attractant-free, modified bednet traps that target free-flying mosquitoes. The trap is divided diagonally to give 4 quadrants, which allows for the determination of mosquito flight direction. Mosquitoes were collected by aspiration every hour from 18:00 h to 06:00 h from each quadrant.

Mosquitoes sampled from all traps (*n *= 19,467) were morphologically identified in the field to species, group, or complex level [28]. Anophelines were individually stored on desiccant and shipped to the University of Notre Dame, USA, for molecular identification and data analysis.

### Molecular processing and sequence analysis

A subset of morphologically identified mosquitoes (*n* = 4102 out of 24,509) were sequenced at the ribosomal DNA internal transcribed spacer region 2 (ITS2) and/or cytochrome *c* oxidase subunit 1 (*cox*1) loci towards species identification [24, 26]. Samples were first sequenced at the ITS2 locus, and then a subset of samples with successful ITS2 sequences were also sequenced at the *cox*1 locus. Samples sequenced were randomly chosen across trapping method and year collected. Approximately 10% of samples from each trap type were randomly selected and sequenced. Only 4.5% of the samples from barrier screens with eaves were randomly selected and sequenced due to collections only being performed during two collection periods.

### Species identification

Molecular identification was conducted blind to morphological identity to prevent any bias in the analysis. Final species confirmation required high sequence identity (≥ 98%) to voucher sequences in multiple databases [24–26, 31, 32]. *cox*1 and ITS2 database comparisons for each sample were paired to determine species when either *cox*1 or ITS2 alone did not produce significant results to voucher sequences [24–26, 31, 32]. Consensus sequences were manually inspected for insertions, deletions, and repeat regions to ensure these sequence differences did not inflate divergence and decrease identity scores. Consensus sequences of each sequence group were compared (BLASTn) to the NCBI nr and BOLD databases to identify species. Sequences mapping to the Funestus Group, Hyrcanus Group, Barbirostris Complex, Sundaicus Complex and Maculatus Group were compared to voucher sequences and primers used in diagnostic PCR [17, 33–37].

### Phylogenetic analysis

For *cox*1, 2034 sequences were queried against the nucleotide non-redundant database (date of download: 31 January 2018) using the BLASTn algorithm in the BLAST + v.2.2.18 command line application [38]. Sequences with a percent identity greater than 90% over 75% of the query length matching any *Anopheles cox*1 were retained for further analysis. This resulted in 2013 *cox*1 sequences.

*cox*1 sequences were aligned using MAFFT v7.394 [39] with parameters: –auto –quiet –preserve-case. Aligned sequences were formatted into a nexus alignment using seqmagick (https://fhcrc.github.io/seqmagick/). Nexus-formatted sequences were used to create a haplotype network in PopArt [40] with the inclusion of a trait file indicating the identity of each sequence from the previous BLAST query. PopArt parameters were selected to construct a minimum spanning network with an epsilon value of 0. The final network nodes were colored to reflect species identity.

The program ABGD [41] was used to identify molecular operational taxonomic units (MOTU) for delineating species. Values of intraspecific genetic diversity of 0.03 and interspecific genetic diversity of 0.10 were used to separate clusters. After 10 permutations, 14 clusters were identified following the methods in Puillandre et al. [41]. From each cluster, 2 sequences were randomly chosen for phylogenetic analysis. A phylogenetic tree was constructed in Geneious v 11.1.2 using PhyML with 100 bootstrap samples [42].

A phylogenetic analysis was performed on ITS2 consensus sequences [43]. A multiple alignment of consensus ITS2 sequences was performed with MUSCLE v3.8.31 [44]. After alignment, Gblocks v0.91b was used to eliminate poorly aligned positions and divergent regions [45]. The phylogenetic tree was reconstructed using the maximum likelihood method implemented in the PhyML v3.1/3.0 aLRT program [42, 46] was used to calculate the unrooted tree. Reliability for internal branch was assessed using the bootstrapping method (100 bootstrap replicates). Graphical representation and edition of the phylogenetic tree were performed with TreeDyn v198.3 [47].

## Results

Approximately 24,509 morphologically identified *Anopheles* mosquitoes were sampled in a comprehensive trapping effort. Sampling over almost 3 years ensured the capture of seasonal population variations. Trapping habitats included inside and outside domestic spaces, open fields, rice fields, farms, as well as areas that faced the jungle, marshes, river and mountains. Collections using several different methods ensured that multiple behaviors were solicited in mosquito capture. These included HLCs, both inside and outside houses, capturing human seeking mosquitoes. Barrier screens and Kelambu traps were placed to capture free-flying mosquitoes flying to oviposition sites (forest, river, marsh and ponds) as well as towards multiple hosts (humans, goats, cows and chickens). A set of 4102 specimens, randomly sampled over all trapping types, spaces and periods were processed molecularly.

### Molecular species identification

ITS2 and *cox*1 sequences representing 2616 and 2163 *Anopheles* mosquitoes, respectively, were processed. Approximately 677 specimens were sequenced for both ITS2 and *cox*1 loci while the remaining had only one sequence (either ITS2 or *cox*1).

ITS2 sequences representing 2616 *Anopheles* mosquitoes were aligned into 18 sequences with a stringency of greater than 98% identity within each sequence (Table 2). The *cox*1 sequences representing 2163 *Anopheles* mosquitoes and 19 morphologically identified species were aligned into 20 distinct sequence groups (Table 2). The parameters used to produce the final sequence groups resulted in no hyper-variable regions present in any specific sequence group. Similarly, there was little (under 2%) variation in sequences within a sequence group, i.e. none with insertions or deletions more than 2 base pairs (bp). Distinct sequence groups were arbitrarily called sequence groups (AN) 1 through 18 prior to a more in-depth database comparison and species level identification. High similarity (≥ 98% identity), the presence of voucher specimens in multiple databases, concordant ITS2-*cox*1 pairs, and/or sequence alignment to those used in PCR diagnostic assays allowed the identification of eleven species: *An. aconitus* (ANI); *An. karwari* (AN2); *An. peditaeniatus* (AN3); *An. vagus* (AN4 and AN5); *An. barbirostris* (AN6); *An. tessellatus* (AN7); *An. nigerrimus* (AN8); *An. flavirostris* (AN9), *An. crawfordi* (AN10); *An. maculatus* (AN11); and *An. kochi* (ANI2) (Table 2).

**Table 2:** Overview of molecular identifications

Sequence group	No. of samples (ITS2; *cox*1)	Sequence length in bp (ITS2; *cox*1)	ITS2 homology (%ID)	*Cox*1 homology (%ID)	Final ID
AN1	23; 20	515; 651	*An. aconitus* (98.6)	*An. aconitus* (95.9)	*An. aconitus*
AN2	1; 1	495; 327	*An. karwari* (98.7)	*An. karwari* (97.7)	*An. karwari*
AN3	113; 29	524; 698	*An. peditaeniatus* (100)	*An. peditaeniatus* (99.5)	*An. peditaeniatus*
AN4	29; 29	658; 697	*An. vagus* (98.6)	*An. vagus* (96.6)	*An. vagus*
AN5	275; 35	640; 697	*An. vagus* (99.8)	*An. vagus* (96.6)	*An. vagus*
AN6	1305; 222	1470; 704	*An. barbirostris* (97.2)	*An. barbirostris* (98.6)	*An. barbirostris*
AN7	35; 28	592; 643	*An. tessellatus* (96.8)	*An. tessellatus* (95.6)	*An. tessellatus*
AN8	233; 43	592; 652	*An. nigerrimus* (96.7)	*An. nitidus* (96.1)	*An. nigerrimus*
AN9	47; 39	503; 628	*An. flavirostris* (99.8)	*An. flavirostris* (98.9)	*An. flavirostris*
		503; 643		*An. flavirostris* (98.95)	
AN10	5; 4	534; 630	*An. sinensis* (97.9)	*An. crawfordi* (97.1)	*An. crawfordi*
AN11	28; 25	450; 643	*An. maculatus* (95.4)	*An. maculatus* (95.9)	*An. maculatus*
AN12	7; 4	491; 631	*An. kochi* (99.4)	*An. kochi* (100)	*An. kochi*
AN13	32; 29	617; 679	*An. saeungae* (9.0)	*An.* sp. 14 (91.7)	Myzorhynchus Series
AN14	140; 40	420; 689	*An. bancroftii* genotype B (93.3)	*An. coustani* (92.1)	Myzorhynchus Series
		420; 636		*An. farauti* (92.3)	Neomyzomyia Series
AN15	5; 4	381; 689	*An. bancroftii* genotype B (91.5)	*An. coustani* (92.0)	Myzorhynchus Series
AN16	1; 1	615; 616	*An. subpictus* (78.6)	*An. albitarsis* E (91.5)	Genus *Anopheles*
AN17	277; 72	569; 717	*An. sundaicus* (80.7)	*An. albitarsis* (91.4)	Genus *Anopheles*
AN18	60; 52	589; 689	*An. tessellatus* (88.1)	*An. lutzii* (92.0)	Genus *Anopheles*

Table represents the 2616 total ITS2 sequences and the corresponding 677 *cox*1 sequences. Final species identifications are based on both ITS2 and *cox*1 comparisons.  % ID is the percentage identity based on BLAST database comparison. Final species identification, when not to specific species, was based on the lowest common taxonomic identity for the paired ITS2 and *cox*1 sequences

Sequence comparisons of AN1 ITS2 to those used to develop PCR diagnostic assays for the Funestus Group [33] confirmed the identification of *An. aconitus*. Sequence comparisons of AN3, AN8 and AN10 to the species diagnostic sequences and primers [34] further clarified species identities. AN3, AN8 and AN9 were confirmed to be *An. peditaeniatus*, *An. nigerrimus* and *An. flavirostris*, respectively. Even though the *cox*1 sequence had the highest match to *An. nitidus* for AN8, we have used the ITS2 result for the species identification as the species diagnostic PCR would be positive for this species. The AN10 *cox*1 sequence aligned with primers and sequence confirming *An. crawfordi*. However, the AN10 ITS2 top database hit was to a sequence identified as *An. sinensis*, also identical to the *An. crawfordi* sequence in the Hempolchom PCR diagnostic assay [34]. We have designated AN10 as *An. crawfordi* using the results from this PCR, along with support from the *cox*1 hit indicating *An. crawfordi*. The AN11 ITS2 demonstrated the highest similarity to specimens used in the Maculatus Group diagnostic assay [37]. In addition, comparison of these sequences to those in Ali et al. [36] and Garjito et al. [48] demonstrated a 100% match thus confirming *An. maculatus* with the added support from the *cox*1 sequence (Table 2).

Several specimens with matched ITS2 and *cox*1 sequences had homology to different species in the database resulting in uncertain species identification. A conservative approach was used resulting in sequence groups AN14 and AN15 being identified to the lowest common taxonomic level based on the closest identified sequences, instead of a specific species.

Sequence groups AN6 and AN13 both had similarity to the Barbirostris Complex. The ITS2 and *cox*1 sequences of AN6 demonstrated high similarity to *An. barbirostris*. The ITS2 sequence was compared to voucher sequences and primer sites used in Brosseau et al. [17], as the highest homology was to these sequences. Although most similar to *An. barbirostris*, the presence of multiple SNPs, insertions and deletions would result in only a single primer being able to bind–rendering this multiplex assay producing no bands for these specimens.

The AN13 ITS2 sequence was closest to *An. saeungae* with the caveat that the similarity was only in the coding region (9% ID overall and 91% ID to the coding region) with no real homology seen in the intergenic spacer region rendering the Brosseau et al. [17], multiplex PCR incompatible. The *cox*1 sequence was closest to an unknown *Anopheles* species (92% ID) found in both Zambia and the Kenyan highlands. This may represent a new member of the Barbirostris Group but due to the conservative approach to identification and high percentage identity requirement for both ITS2 and *cox*1 loci, these sequences were identified to the Myzorhynchus Series.

Sequence groups AN16-AN18 did not share greater than 93% identity (when combined with sequence coverage) with any nr database sequences and were identified to the genus level only. The comparison of ITS2 sequences from AN16 and AN17, to those used to develop the Dusfour et al. [35], diagnostic PCRs, revealed the absence of any primer sites with low homology across the sequences. The combination of disparate ITS2 and *cox*1 sequence pairs also complicates this analysis. AN16, AN17 and AN18 remain unknown species.

### Phylogeny

**Fig. 2:**
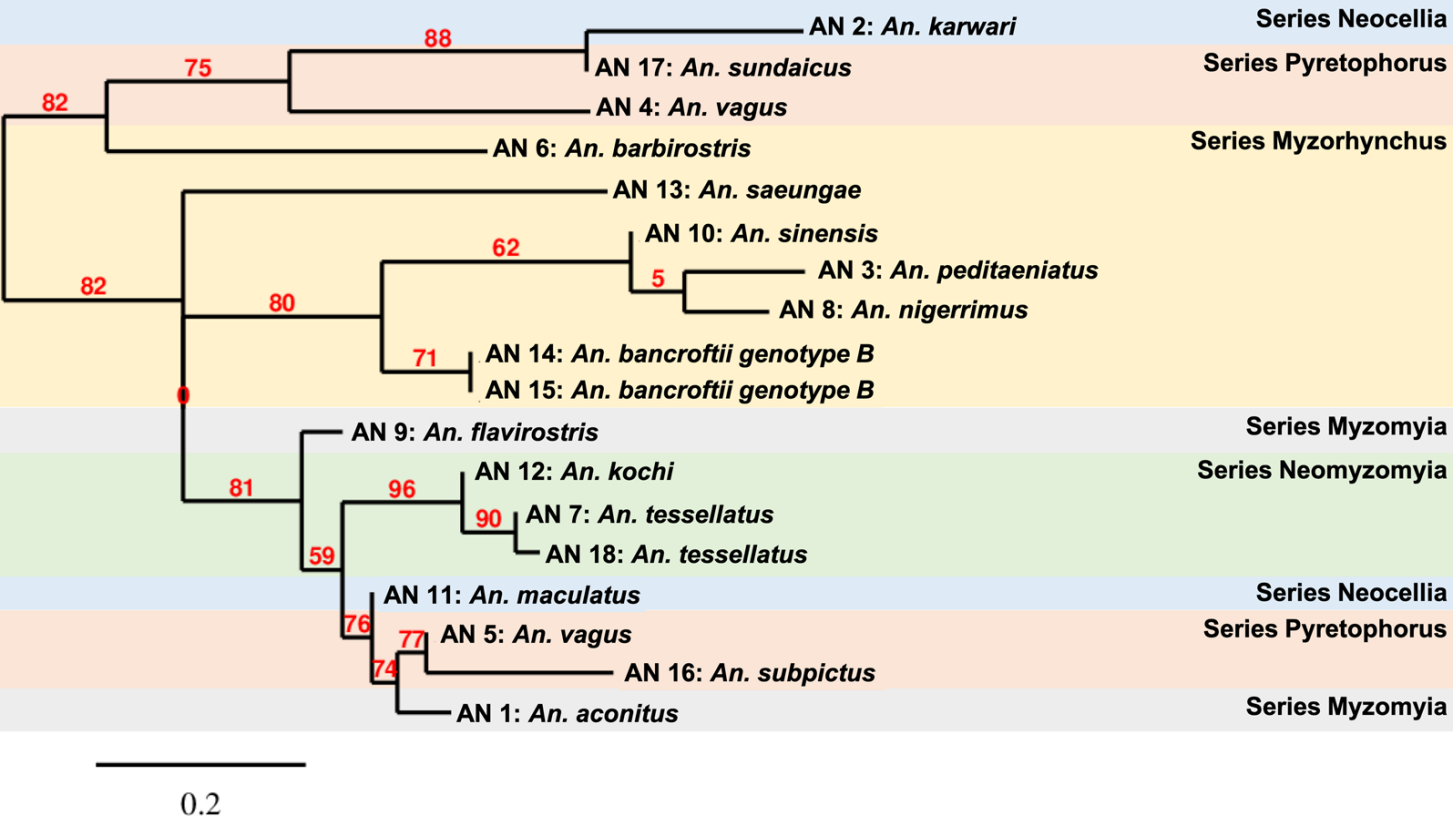
Phylogenetic tree based on ITS2 sequences. A phylogenetic tree was constructed using 1985 ITS2 sequences in Geneious v 11.1.2 using PhyML with 100 bootstrap replicates. Bootstrap values are shown as percentages above the branches

Consensus ITS2 sequences were aligned to construct a phylogenetic tree. The ITS2 tree (Fig. 2) groups putative species as expected based on their taxonomy. The *An. barbirostris* sequence groups (AN6 and AN13), *An. nigerrimus* (AN8), *An. sinensis* (AN10), *An. peditaeniatus* (AN3), and *An. bancroftii* sequence groups (AN14 and AN15) clustered separately as part of the Myzorhynchus Series. Members of the Barbirostris Complex, *An. nigerrimus*, *An. sinensis*, *An. peditaeniatus*, and *An. bancroftii* group all belong to the subgenus *Anopheles*, whereas the rest of the species identified here are members of the subgenus *Cellia*. *Anopheles vagus*, *An. subpictus* and *An. sundaicus* (i.e. AN4, AN5, AN16 and AN17, respectively) clustered together as part of the Pyretophorus Series. *Anopheles vagus* AN4 was more closely related to *An. sundaicus* AN17 while *An. vagus* AN5 is more closely related to *An. subpictus.* The Myzomyia Series cluster was composed of *An. aconitus* and *An. flavirostris* (AN1 and AN9, respectively) while the Neocellia Series cluster was composed of *An. karwari* and *An. dispar* (AN2 and AN11, respectively). *Anopheles tessellatus* and *An. kochi* clustered as part of the Neomyzomyia Series (AN7, AN12 and AN18, respectively). Species AN12-AN18 had low similarity (< 93% ID) to known sequences in the database and may represent new species.

**Fig. 3:**
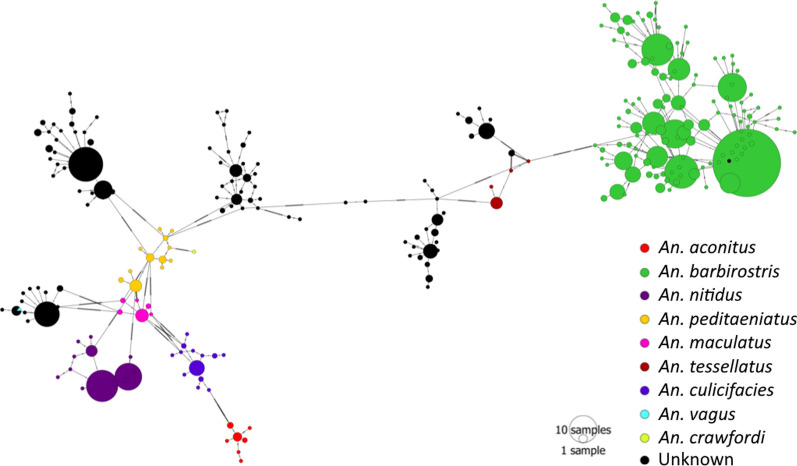
Haplotype network based on 2034 *cox*1 sequences from this study. Each observed haplotype is indicated by a filled circle, sized according to its frequency and colored according to the *Anopheles* species represented. Haplotype relationships are indicated by lines; mutational steps between haplotypes are represented by the number of lines

Consensus *cox*1 sequences were aligned to construct a haplotype network (Fig. 3). Among these *cox*1 samples, 9 haplotypes allowed species identification: *An. aconitus*; *An. barbirostris*; *An. nitidus*; *An. peditaeniatus*; *An. maculatus*; *An. tessellatus*; *An. culicifacies*; *An. vagus*; and *An. crawfordi*. Furthermore, black circles represent possible cryptic species in the dataset, numbering 5 to 8 species. The network indicates there is intermediate divergence between *An. barbirostris* and *An. culicifacies* nodes.

### Comparison of molecular and morphological identifications

Morphologically derived species identities revealed that no species were correctly identified with 100% accuracy when compared with molecular identifications (Table 3). Morphological identifications had the highest rate of accuracy for the most abundant species in the area, *An. barbirostris* and *An. vagus*, 92.1% and 87.6%, respectively, when compared to molecular analysis (Table 3). For the remaining four distinct sequences molecularly identified to species, the percentage of correctly identified morphological specimens ranged from 0% to 83% (Table 3). Sequencing demonstrated the presence of 18 distinct sequence groups while morphology indicated the presence of 19 species. Specimens morphologically identified as *Aedes albopictus*, *An. barbumbrosus*, *An. hyrcanus*, *An. indefinitus*, *An. parangensis*, *An. pseudobarbirostris*, *An. schueffneri*, *An. sulawesi*, *An. umbrosus* and *Culex* spp., did not have sequences that reflected these identifications (Table 3). Of these 10 species, 6 have ITS2 and/or *cox*1 sequences in the databases indicating that these morphological results were probably misidentifications. Morphological identification did not identify any *An. karwari*, *An. peditaeniatus* or *An. sinensis* specimens, all identified molecularly. Members of sibling species cannot be differentiated morphologically; so, the specimens morphologically identified as *An. barbirostris* were considered correct since molecular identification indicating these as being in the Barbirostris Complex.

**Table 3:** Molecular identification of species employing both ITS2 and *cox*1 comparisons

Molecular species ID	Morphology species ID	No. of sequences identified molecularly	No. of correctly identified specimens based on morphology	% of correctly identified specimens based on morphology
*An. aconitus*^a^	a, b, c, e, r	28	8	28.6
*An. barbirostris*^a^	c, d, f, g, j, k, p, q, r, x	2396	2207	92.1
*An. karwari*^a^	m	1	0	0
*An. peditaeniatus*^a^	c, d, f, j, k, q, x	133	0	0
*An. tessellatus*^a^	b, c, d, p	41	34	83.0
*An. vagus*^a^	c, d, g, j, k, p, r	331	290	87.6
Total	**–**	2931	2539	86.6

^a^Species previously confirmed as malaria vectors in Indonesia

Morphologically-based species identifications included: **a***Aedes albopictus*; **b***Anopheles aconitus*; **c***An. barbirostris*; **d***An. barbumbrosus*; **e***An. flavirostris*; **f***An. hyrcanus*; **g***An. indefinitus*; **h***An. kochi*; **i***An. maculatus*; **j***An. nigerrimus*; **k***An. parangensis*; **l***An. pseudobarbirostris*; **m***An. schueffneri*; **n***An. subpictus*; **o***An. sulawesi*; **p***An. tessellatus*; **q***An. umbrosus*; **r***An. vagus*; **x***Culex*. Number and percentage of correctly identified morphological specimens are calculated from the number of sequences that were molecularly identified per species

## Discussion

For Indonesia to achieve its malaria elimination goal, intervention strategies need to cater to varied and complex transmission dynamics with multiple local mosquito vectors. Understanding temporal vector compositions along with their bionomic traits may allow for better and more targeted intervention strategies as well as understanding important gaps in protection.

This study represents an initial foray into characterization of the *Anopheles* species in a single village within an area known for its biodiversity [5, 10–13]. Multiple trapping methods were utilized over multiple seasons and years to ensure capture of as many species as possible. Eighteen separate species sequences were identified in this single 1 km^2^ area over the span of almost 3 years. Molecular identification using both ITS2 and *cox*1 sequences was used for species identification with the conservative algorithm outlined. The unexpected high number of novel sequences, combined with ITS2 and *cox*1 sequences from the same specimen matching separate (though closely related species) in the database, resulted in multiple specimens from each sequence group being re-sequenced to eliminate the possibility of contamination or mislabeling.

Of the molecularly identified sequences, only a subset could be identified to species based on present available data and the conservative algorithm for species identification. Of these molecularly identified species, ten are previously confirmed malaria vectors from Indonesia: *An. aconitus*; *An. barbirostris*; *An. karwari*; *An. peditaeniatus*; *An. tessellatus*; *An. vagus*; *An. kochi*; *An. flavirostris*; *An. nigerrimus*; and *An. maculatus* [12, 13, 49–55]. Although these Barbirostris Complex species have unclear vector status, they are likely vectors since specimens identified morphologically as *An. barbirostris* have been considered medically important vectors in Sulawesi [12, 18]. Although it was impossible to identify to the species level the other molecularly identified sequences, their likelihood of containing confirmed vectors is plausible. The diversity of malaria vectors in Indonesia with suboptimal morphological identifications highlights the importance of integrating molecular identification into vector studies.

*Anopheles vagus* has been previously suspected of being a species complex [12]. In Timor-Leste on Timor Island, an island south of Sulawesi, a putative species *An. vagus* genotype B has been found positive with *Plasmodium* CS protein [56]. Furthermore, two forms of mitotic karyotypes have been found from *An. vagus* in Thailand [57], indicating intraspecies. However, further studies were unable to determine if the two forms were sibling species [58]. In this study, ITS2 sequence groups indicated two distinct groups identified as *An. vagus* (AN4 and AN5). Additionally, the ITS2 phylogenetic tree indicates that there are two genetically distinct *An. vagus*-like species (Fig. 2). These results corroborate the previous literature elucidating that *An. vagus* is likely a part of a species complex.

Sequence groups AN6 and AN13, both members of the Barbirostris Complex, demonstrate the complexity present in this group of species. Comparisons to multiple databases, voucher specimens and species identification assays [17] resulted AN6 being identified as a sequence variants of *An. barbirostris*. These differences in the non-coding ITS2 spacer region in AN6 may be reflective of the Sulawesi island population of *An. barbirostris*, supported by the high *An. barbirostris* homology with the *cox*1 sequence.

Sequence group AN13 had low ITS2 homology (9%) to known members of the Barbirostris Complex. This is not surprising, since previous studies have indicated that the Barbirostris Subgroup ITS2 region is large in size and has internal repeats, characteristics that make species-diagnostic PCR based on ITS2 difficult [17, 23]–also relevant to AN6. Furthermore, the 16 corresponding *cox*1 sequences identified AN13 as an unknown *Anopheles* species (92% ID) demonstrating closest similarity to an unknown African species previously reported from the Western Kenyan Highlands and Zambia [24, 26]. The combination of the novel ITS2 spacer region combined with a *cox*1 sequence closest to an unknown African species points to a novel species in this complex.

Similar results were seen for sequence groups AN10 and AN11 in which ITS2 and *cox*1 results had differing sequence homology with high percentage identity while similar associated ITS2 and *cox*1 sequences were present in the database. Comparison to voucher specimens and primers in diagnostic PCRs [34, 36, 37] enabled their species identification (Table 2). The sequence differences observed with those in PCR diagnostics as well as those in the NCBI database highlight how morphological misidentification may perpetuate species level misidentifications even in published databases.

All ITS2 and *cox*1 sequences with conflicting, high percentage identification are closely related taxonomically and the discrepancies may be due to variation in sequences based on these collections being from both a center of biodiversity as well as being an isolated island population and therefore, having diverged from other populations represented more frequently in the databases. Note that multiple randomly chosen samples from each of these groups were re-sequenced to confirm the results. For example, the majority of the research that focuses on the Funestus Group is concentrated in sub-Saharan Africa [59–61]. Furthermore, the sequences within each of these species may vary with the island of Sulawesi being a biodiversity hotspot, further complicating analyses. This study supports the likelihood that distributions and phylogenetic relationships between species in the Barbirostris Subgroup, Nigerrimus Subgroup, Hyrcanus Group and Maculatus Group, need further clarification and research. Therefore, additional research that implements nuclear and mtDNA sequencing within in Indonesia is necessary to accurately identify species that are malaria vectors.

Six ITS2 and paired *cox*1 sequences (AN13-AN18) could not be identified to species or species group because homology did not meet the conservative criteria (< 93% identity) when comparing to the databases [32, 62]. This may be due to these species not having the related sequences in the database, further stressing the need for more molecular analysis to be completed in areas with high diversity and unknown species composition. It is possible that the species have diverged sufficiently because Karama is an isolated habitat on a biodiverse island–Sulawesi. Furthermore, to the best of our knowledge, this study represents a first-time sample and molecular identification for any Sulawesi population. These six unidentified sequences may represent divergence from specimens in database, novel and/or unidentified sibling species, subspecies, and/or cryptic species.

This study highlights the importance of cross-referencing morphological identifications with molecular identifications, especially in areas of high vector diversity. Morphological identifications were most accurate, when compared to molecular identifications, for the most abundant species groups in the area, *An. barbirostris* and *An. vagus* (92.1% and 87.3%, respectively). However, when examining less common species, a comparison of molecularly and morphologically derived species identities demonstrated the inconsistency of relying solely on morphological identification. Finally, all molecularly identified species were mistaken for multiple species when utilizing morphological identification alone. Misidentifications resulting from morphological identification may have negative downstream effects when determining species’ bionomic traits, associations of vector status, entomological inoculation rates, and impacts on control [31]. The discrepancy between morphological and molecular identification underlies the importance of incorporating molecular tools to help distinguish vector species.

## Conclusions

This study highlights the importance of cross-referencing morphological identifications with molecular identifications to determine mosquito species composition. Eleven distinct sequences were identified to species, with an additional seven sequences identified to either subgroup, group, or series. Three sequences could only be identified to the genus level, as the percentage identification was too low to identify them to a series. This is the first study to characterize species composition in Karama, West Sulawesi with molecular identification techniques. Future studies employing sequencing are required to clarify the species in several taxonomic groups, as well as their distributions and vector status. Identifying the primary and secondary malaria vectors in this area is vital for appropriate, targeted malaria control interventions and accurate monitoring of their effectiveness. Finally, this study design and analysis represents a dataset and methodologies that can be applied anywhere to enable Indonesia to move forward with their goal of malaria elimination.

## Data Availability

Data supporting the conclusions of this article are included within the article. Representative sequences were deposited in the GenBank database under the accession numbers MT740899-MT740916 (ITS2) and MT753033-MT753050 (*cox*1). The raw datasets used and/or analyzed during the present study are available from the corresponding author upon reasonable request.

## Abbreviations

WHO: World Health Organization
PCR: Polymerase chain reaction
HLC: Human landing catch
